# Monte Carlo Simulation of Diffuse Optical Spectroscopy for 3D Modeling of Dental Tissues

**DOI:** 10.3390/s23115118

**Published:** 2023-05-27

**Authors:** Mousa Moradi, Yu Chen

**Affiliations:** 1 Department of Biomedical Engineering, University of Massachusetts, Amherst, MA 01003, USA; mousamoradi@umass.edu; 2 Institute for Applied Life Sciences, University of Massachusetts, Amherst, MA 01003, USA

**Keywords:** Monte Carlo, DOS, 3D tooth model, transmittance, reflectance

## Abstract

Three-dimensional precise models of teeth are critical for a variety of dental procedures, including orthodontics, prosthodontics, and implantology. While X-ray-based imaging devices are commonly used to obtain anatomical information about teeth, optical devices offer a promising alternative for acquiring 3D data of teeth without exposing patients to harmful radiation. Previous research has not examined the optical interactions with all dental tissue compartments nor provided a thorough analysis of detected signals at various boundary conditions for both transmittance and reflectance modes. To address this gap, a GPU-based Monte Carlo (MC) method has been utilized to assess the feasibility of diffuse optical spectroscopy (DOS) systems operating at 633 nm and 1310 nm wavelengths for simulating light-tissue interactions in a 3D tooth model. The results show that the system’s sensitivity to detect pulp signals at both 633 nm and 1310 nm wavelengths is higher in the transmittance compared with that in the reflectance mode. Analyzing the recorded absorbance, reflectance, and transmittance data verified that surface reflection at boundaries can improve the detected signal, especially from the pulp region in both reflectance and transmittance DOS systems. These findings could ultimately lead to more accurate and effective dental diagnosis and treatment.

## 1. Introduction

Accurate three-dimensional (3D) models of teeth are essential for various dental applications, including orthodontics, prosthodontics, and implantology [1,2]. These models can aid in diagnosis, treatment planning, and the fabrication of dental prostheses. Various imaging techniques are used to obtain 3D data of teeth, including X-ray-based imaging devices and optical devices [3,4]. While X-ray-based imaging devices provide excellent resolution, they expose patients to ionizing radiation, which can be harmful. In contrast, optical devices such as structured light scanners and intraoral scanners offer a non-invasive and radiation-free method of acquiring 3D data of teeth [5]. Diffuse optical spectroscopy (DOS) has also been used for various medical applications, including the detection of dental caries and erosion [6,7,8] while it does not involve any harmful radiation. Early detection of caries is crucial to prevent further damage to the tooth structure. However, further research is necessary to verify the effectiveness of optical devices such as DOS in creating precise 3D models, and to develop more advanced imaging systems suitable for clinical applications.

Monte Carlo (MC) simulation is a well-established method for modeling light interaction in biological tissues [9,10,11,12]. It is widely used in the field of optics and biophotonics for simulating the propagation of photons through different biological media [12,13]. MC simulations are considered the benchmark for emulating actual optical devices due to their ability to accurately regulate all the parameters associated with the process of photon migration through turbid media, such as multiple scattering, scattering anisotropy, absorption, reflection, and refraction [9,10,14]. Therefore, to create a precise model of the interactions between light and human teeth, it is essential to comprehend the fundamental principles of light scattering, absorption, source-detector separations (SDSs), and tooth geometry. These parameters rely on the specific optical wavelength, sensor geometry, and the precision of the 3D model to be implemented.

The current available literature on the interaction between light and tissue in 3D teeth models is limited. In 1995, Vaarkamp et al. [15] used analytical solutions such as the Beer-Lambert law (BLL) to detect approximal caries lesions in premolar teeth at 633 nm wavelength. This research indicated that the radiance change caused by a caries lesion is mainly determined by the enamel region lesion part. In 2011, Fu et al. [11] created an MC technique for a 3D tooth model that could be used in photodynamic therapy (PDT) to estimate the amount of light energy deposited in the target area of the tooth. They claimed that the more accurate geometry and optical properties of the tooth, the more accurate estimation for the dosage. Shi et al. [16] in 2013 developed a MC simulation for imaging human teeth by modeling the optical coherence tomography (OCT) signal. They used a gaussian beam emitting light at a 1310 nm wavelength. Their results showed that the OCT signal decreases significantly with increasing mineral loss and the best focus position was located below and close to the enamel region. Gawad et al. [17] in 2017 used a MC model to simulate a laser diffuse reflectance setup for identifying the optimum wavelength as well as SDS position to achieve the highest detected signal. They found that red light with a wavelength of 635 nm is likely the most secure visual color to use in the oral cavity. In 2018 Jayasankar et al. [18] utilized MC simulations of light transport in a two-layered tooth to implement Raman scattering. Their simulations examined the optimal illumination pattern for the detection of the dentin region in the tooth, using photons launched as a pencil beam or broad beams of radii 0.1 cm, 0.2 cm, and 0.3 cm.

Previous computational and MC models of teeth, which depended on BLL, basic diffusion theory, or random walk theory, were not enough to investigate the interactions between light and heavily scattering and absorbing tissue mediums. As of now, there is no existing research that demonstrates optical interactions with all compartments of dental tissue utilizing both reflectance and transmittance modes.

This study, aimed at assessing the feasibility of using MC simulation to model light-tissue interactions in a three-compartments teeth model for DOS systems operating at 633 nm and 1310 nm wavelengths. The 633 nm wavelength was used in some dental applications such as light dosimetry analysis [11] and fluorescence imaging [15]. The 1310 nm wavelength was also used in dental imaging such as near-infrared transillumination [19], OCT [16], and polarization sensitive OCT techniques (PS-OCT) [20], to detect interproximal caries [21,22], occlusal caries [19], and depth-resolved images of teeth [16,23]. These techniques can provide information about the internal structure of teeth and detect early carious lesions. 

The main contributions of this paper can be summarized as follows: (1) unlike previous work, this research primarily provides a novel and realistic mesh-based approach for modeling 3D molar tooth geometry using MC modeling for DOS systems operating at 633 nm and 1310 nm wavelengths. This approach has the potential to offer more precise results compared to the homogeneous and monolayer approaches commonly employed for teeth models [11,17]; (2) the effect of reflection at boundaries on DOS systems in both reflectance and transmittance geometries have been investigated at 633 nm and 1310 nm. By simulating the optical properties of teeth at these wavelengths, researchers and clinicians can gain insights into the interactions between light and dental tissues, optimize the design of optical devices for dental imaging and diagnostics, and improve the accuracy and effectiveness of dental treatments.

## 2. Materials and Methods

### 2.1. Model Geometry and Optical Properties of Tooth

**Figure 1:**
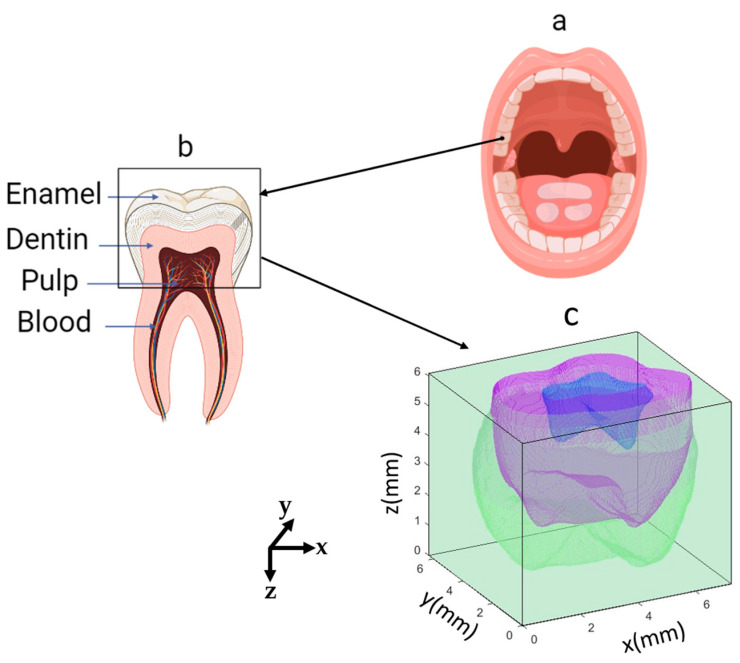
The location of the second molar tooth tissue (**a**), a zoomed view of different tissue (**b**), and the upside-down view of the developed meshed-based model for this tooth (**c**). The box in (**b**) indicates the crown region of the tooth. Different colors in (**c**) correspond to the different tissue types (blue: pulp, purple: dentin, green: enamel).

Figure 1 shows the anatomical characteristics of the dental tissue model. The second molar tooth geometry (Figure 1a) was obtained from the literature [24]. The thickness range of the second molar tooth in the upper jaw for adults is 5.7–8.3 mm [24]. To simulate real optical devices, a 6 mm thickness for the crown has been assumed and the source and detector will be located on it. To ensure that the developed model is precise enough to replicate the features of a real tooth, over 2 million voxels were utilized in the 3D geometry. Table 1 summarizes the volume fractions of each compartment which was used to build the tooth model.

For the analysis, the 3D molar tooth has been divided into three compartments: enamel, dentin, and pulp regions. The enamel region is the hardest tissue in the dental structure and typically ranges in thickness from 1 to 3 mm from the top surface. The pulp region has only 4% of the total geometry; unlike previous research [11], assigning 10% to blood was not giving strong signals for the developed geometry. Therefore, it has been assumed that the blood content was uniformly distributed in pulp. Pulp is made up of living connective tissue that is situated in the center of the tooth, and it receives a blood supply through the tooth root [7]. The optical properties of enamel and dentin at 633 nm and 1310 nm wavelengths have been extensively researched in previous studies [11,15,22,25]. Due to lack of data at 1310 nm, optical properties of generic connective tissue were used for the pulp region [26,27]. Anisotropic factor (g) and refractive index (n) were obtained from literature [11,22]. Table 2 summarized the optical properties used in this study.

### 2.2. Monte Carlo Set Up

**Figure 2:**
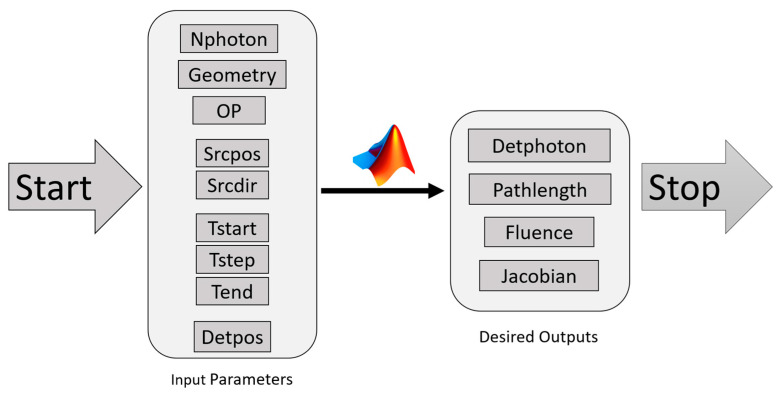
Block diagram for the configured MC model. Optical properties (OP) were obtained as shown in Table 2. Detector position (Detpos) was set horizontally with respect to the source position (Srcpos).

MC Simulation was performed using MATLAB-based Monte Carlo Extreme (MCX) due to its hardware acceleration and heterogeneous media support [10]. Figure 2 displays the block diagram depicting the fundamental procedures of the MC simulation employed to propagate light through dental tissues. The diagram demonstrates that a photon packet was initially launched onto the tissue surface with predetermined direction (Srcdir) and position coordinates (Srcpos). The photon packet had an initial statistical weight of w = 1. The minimum number of simulated photons in this work was $5\times 10^{8}$ to achieve a convergence rate of 0.00004. Equation (1) shows the relationship between the number of input photons (*Nph*) and convergence rate [14]. Both external boundary reflection have been taken into account and a bounding box with high absorption and scattering characteristics. This method assured us that although the majority of scattering and absorption events took place within the bounding box, the overall behavior of photons within each tissue structure could be modeled while reducing the required number of computational iterations. Using this method, 5 iterations were performed to achieve the results. To prevent memory crashes, the simulation was restricted to a time frame of 1 ns (Tend) and utilized 0.1 ns steps (Tstep).

(1) Convergence Rate=1Nph

Following the completion of each iteration, the quantity of photons that were detected by the detector, the optical path length, and the fluence for each photon packet were recorded. When the photon packet intersected with the boundary, an adjustment to determine whether it should internally reflect or transmit through has been made. To calculate the scattering angle of the photons, the Henyey-Greenstein phase function was employed to calculate scattering angle (θ) of each event as shown below:

(2) $$\theta =cos^{-1}\left(\frac{1}{2g}\left[1+g^{2}-{\left(\frac{1-g^{2}}{1-g+2g\delta }\right)}^{2}\right]\right)$$

where g is the anisotropic factor (0 < g< 1) and δ represents a computer-generated pseudo-random number (0 < δ < 1).

A dedicated system consisting of an Intel CPU (with 3.70 GHz and 10 cores) and an RTX-3090 GPU (with 24 GB onboard memory) running on a 64-bit operating system was utilized for the simulation. The MC code was written using MATLAB R2022a (MathWorks, Inc., Natick, MA, USA).

### 2.3. Calculation of Mean Light Distance, Weighted Detected Photons and Scattering Events

Using MC simulations, the distances traveled by photons exiting each tissue type, which was referred to as partial pathlengths (*PP*), were recorded. Then, the mean partial pathlengths (*MPP*) for each tissue type *i* was defined as below [28]:

(3) $$MPP_{i}\left(V_{i,}T\right)=\frac{\sum_{j=1}^{NT}W_{j}PP_{j}\left(V_{i,}T\right)}{\sum_{j=1}^{Ntot}W_{j}}$$

where $V_{i}$ (*i* = 1,2, …, *M*) denote discretized voxels, T is the time gate, $W_{j}$ refers to the detected weights for jth detected photons, *NT* is the index of detected photons during the time gate *T*, and *Ntot* is total detected photons. After obtaining the *MPPs* for every layer, the mean total pathlength (*MTP*) was calculated for an *M*-regions dental tissues by summing up all the *MPPs* together as shown below:

(4) $$MTP=\overset{M}{\underset{i=1}{\sum }}MPP_{i}$$

In each simulation, a Jacobian matrix (also called a spatial sensitivity matrix [29]) was generated. This matrix shows how changes in localized optical properties are related to changes in measurements from specific source-detector pairs. The photon replay algorithm was performed as described in [29] in two steps. Initially, the number of photons that reached the detector was saved in the computer. Then, a second MC simulation was initiated to ensure that the photons followed the same path as before and were captured again. Based on these procedures, the Jacobian matrix (*J*) for absorption and scattering coefficients were obtained as shown below [29]:

(5) $$J_{\mu_{a}\left(V_{i},T\right)}=-MPP\left(V_{i},T\right)$$

(6) $$J_{\mu_{s}\left(V_{i},T\right)}=\frac{\bar{Nsc}\left(V_{i},T\right)}{\mu_{s}\left(V_{i}\right)}-MPP\left(V_{i},T\right)$$

where MPP is the mean partial pathlength (as defined in Equation (3)). By plugging Equation (3) into (5), the total weighted detected photons can be calculated. $\bar{Nsc}$ in Equation (6) denotes the average scattering counts of all photons traversing $V_{i}$ in the time gate T which can be further simplified as shown below:

(7) $$\bar{Nsc}\left(V_{i,}T\right)=\frac{\sum_{j=1}^{NT}W_{j}Nsc_{j}\left(V_{i,}T\right)}{\sum_{j=1}^{Ntot}W_{j}}$$

## 3. Results

In this section, simulation results for the transmittance and the reflectance mode geometries have been reported. In the transmittance mode, the source and detector were placed 7 mm away from each other. The effect of surface reflection on the measurement of spatial sensitivity at the detector has been investigated in two situations: one with surface reflection (WSR) and another without surface reflection (W/OSR) at boundaries. The reflectance mode involved placing source-detector separations (SDSs) at three distinct distances, namely 250 µm, 750 µm, and 1.5 mm. Commercial DOS systems used in dental detection use various probe sizes such as 50 µm, 100 µm, 200 µm, and 400 µm [17,30]. While smaller probe sizes are suitable to detect very fine details and small irregularities, larger probe sizes are typically used for initial screenings. Therefore, the probe size of 200 µm offers a good balance between fine detail detection and broader coverage. Thus, throughout this study, a probe size of 200 µm with a Gaussian beam were chosen.

### 3.1. Detected Photons, Fluence Distribution, and Optical Pathlength

**Figure 3:**
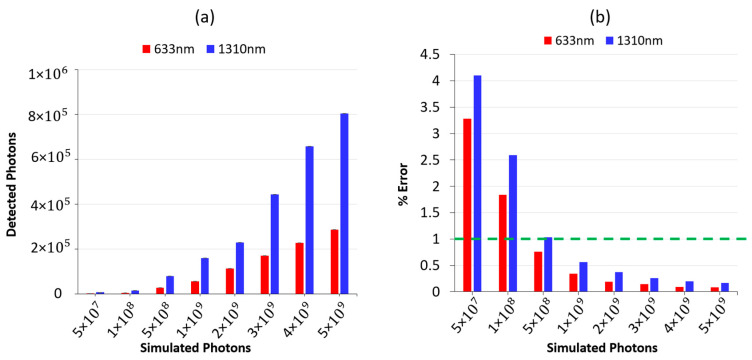
Detected photons by detector for the transmittance mode dental model (**a**), and the calculated errors for each simulation (**b**). The green dashed line is presented in (**b**) to indicate the threshold of a 1% error rate, which can be utilized to determine the suitable number of input photons. Error bars in (**a**) show 1 standard deviation.

To determine the ideal number of input photons for achieving an error rate of 1% or less, the simulation was conducted using a range of (50 to 500) ×$10^{6}$ photons (Figure 3a) then the error rate for each run was calculated by dividing the standard deviation by the mean. Simulation results show that the optimal number of input photons at 633 nm wavelength was found to be $5\times 10^{8}$ photons, whereas for 1310 nm, it was determined that $6\times 10^{8}$ photons were necessary to achieve an acceptable error rate (Figure 3b). Throughout the paper, simulations were carried out using $6\times 10^{8}$ photons for the aforementioned reason.

**Figure 4:**
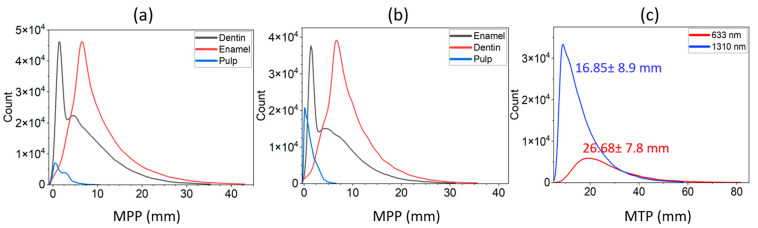
Partial pathlength for each tissue type: at 633 nm (**a**) and at 1310 nm (**b**). The total pathlength is defined as cumulative partial pathlength of all compartments (**c**).

As shown in Figure 4, the distribution of distance that photons can travel in dental tissue between the source and detector in the transmittance geometry has been estimated. Photons that contribute to the measurement were only considered. At 633 nm, the maximum number of photons through pulp spent 3.66 mm (Figure 4a), while at 1310 nm, the maximum occurred at a pathlength of 1.78 mm (Figure 4b). On the other hand, photons traveled the least path through the pulp region compared with enamel and dentin regions. The longer path at both wavelengths was recorded at the dentin region. The cumulative path length at each wavelength (Figure 4c) showed that, for 1310 nm, light can travel the mean distance 16.85 ± 8.9 mm, whereas for 633 nm, the value was 26.68 ± 7.8 mm.

**Figure 5:**
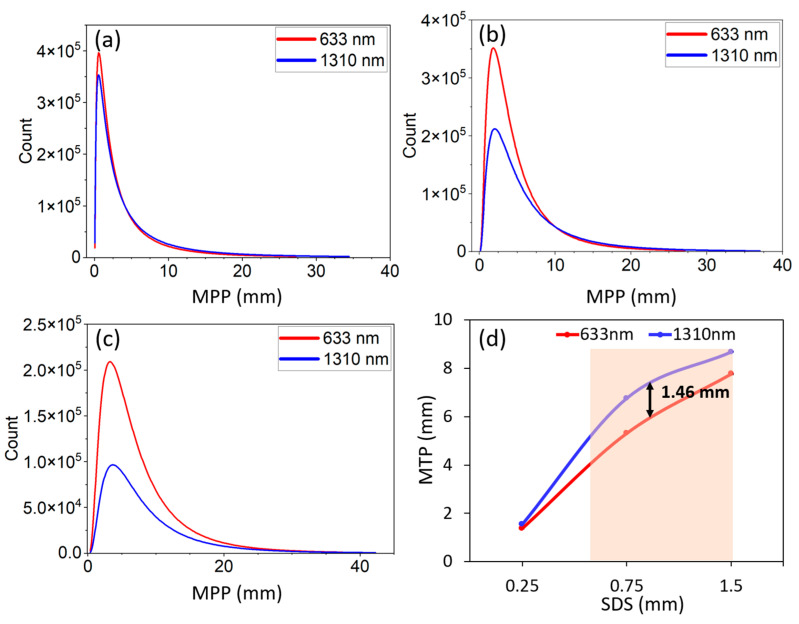
The mean partial pathlength at 0.25 mm (**a**), 0.75 mm (**b**), and 1.5 mm (**c**) SDSs. The peaks of plots a-c represent the maximum number of events. The shaded area in (**d**) represents the region where the pathlengths show a deviation between the two operating wavelengths larger than a 1 mm unit.

Figure 5 illustrates the mean partial pathlength of photons through dental tissue in the reflectance mode. As SDSs increase, the light spends a longer distance in the tissue. At all SDSs (0.25 mm, 0.75 mm, and 1.5 mm), the 1310 nm wavelength traveled a longer distance into tissues than 633 nm (Figure 5a–c). For SDS = 0.25 mm, photons can go through the tooth beyond a pathlength of 1.35 mm. Figure 5d shows that for SDS ≥ 0.6 mm, the pathlength difference between two wavelengths is greater than 1 mm, although they did not show any significant difference (*p*-value > 0.05; Fisher’s exact test). However, at the SDS = 0.75 mm, the maximum deviation in MTP was observed. At 1310 nm, the MTP traveled by photons was 8.75 mm (as shown in Figure 5d) while this value was 7.81 mm for 633 nm.

**Figure 6:**
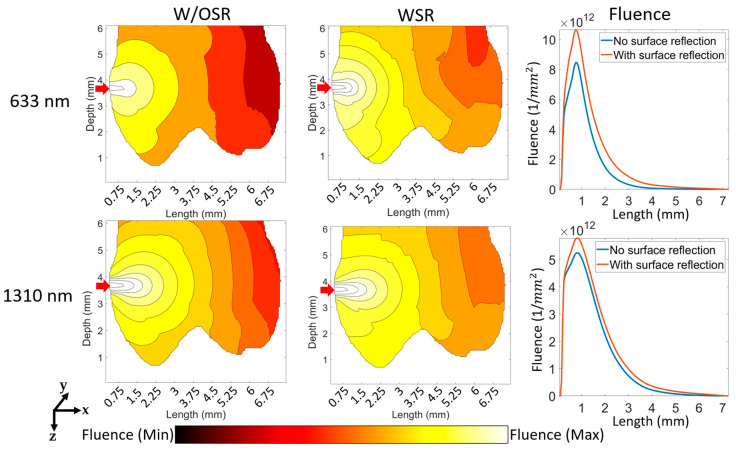
Fluence distribution versus length for optical devices with no surface reflection at boundaries and with surface reflection at boundaries. The labels of ‘633 nm’ and ‘1310 nm’ refer to the respective two rows and “WSR”, “W/OSR”, and “Fluence” refer to the respective three columns. The red arrows show the source position and propagation direction. The scale used for the color bar is logarithmic.

Figure 6 shows the fluence distribution for WSR and W/OSR conditions at 633 nm and 1310 nm wavelengths. At 1 mm distance from source (boundary of enamel and dentin), the fluence reaches a maximum and for this distance, a higher fluence at 633 nm than that at 1310 nm has been observed. Generally, WSR can provide a higher fluence at 633 nm than that at 1310 nm.

### 3.2. Jacobian Analysis

**Figure 7:**
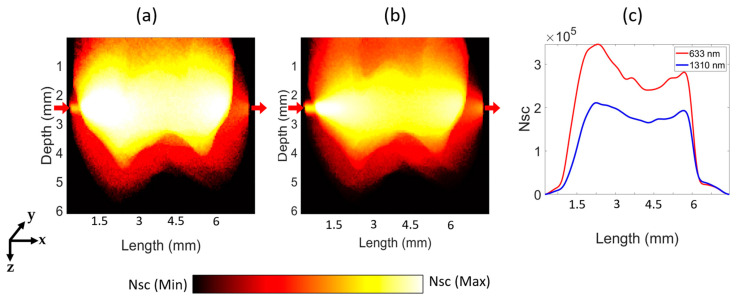
The graphs in (**a**,**b**) illustrate scattering distributions in the transmittance geometry for wavelengths of 633 nm and 1310 nm, respectively. The red arrows in the figures indicate the positions of the optical source and detector from left to right, respectively. The logscale color bar represents the range of scattering events (Nsc) between the maximum and minimum values. Graph (**c**) displays the number of scattering distributions for the dental tissue along the length.

**Figure 8:**
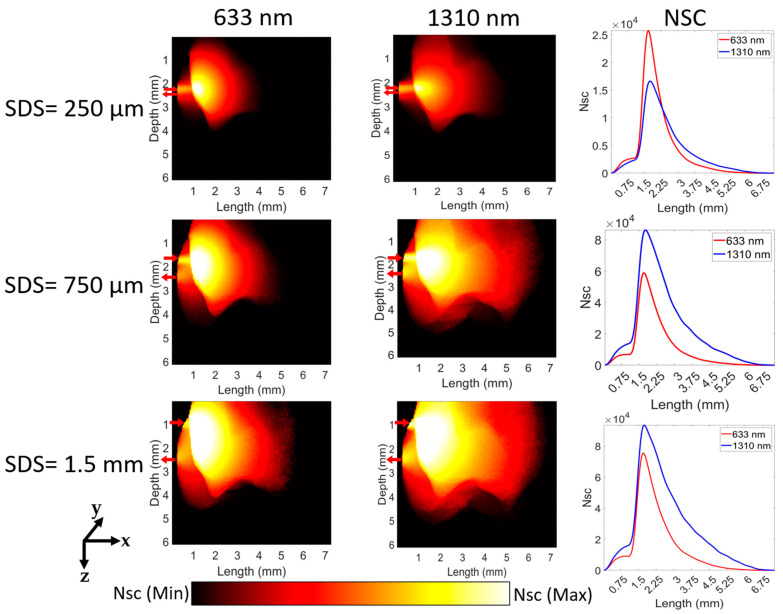
Scattering distribution for the reflectance geometry. The labels of ‘SDS = 250 µm’, ‘SDS = 750 µm’, and ‘SDS = 1.5 mm’ refer to the respective three rows and “633 nm”, “1310 nm”, and “NSC” refer to the respective three columns. The red arrows in each figure show the respective positions of the optical source and detector, pointing towards and away from the figure. The logscale color bar denotes the scattering events (Nsc) within the range of maximum and minimum values. The third column illustrates the quantity of scattering distributions for the dental tissue over a certain distance.

**Figure 9:**
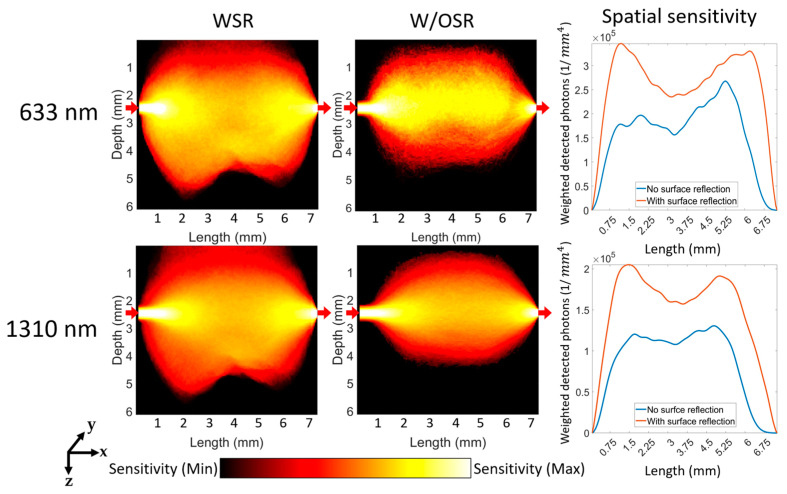
Spatial sensitivity maps for the transmittance geometry at 633 nm and 1310 nm. The labels of ‘633 nm′ and ‘1310 nm′ refer to the respective two rows and “WSR”, “W/OSR”, and “Spatial sensitivity” refer to the respective three columns. The sensitivity of the system was calculated as the integrated weighted detected photons measured at the detector. The scale used for the color bar is logarithmic. The red arrows in the figures indicate the positions of the optical source and detector from left to right, respectively.

**Figure 10:**
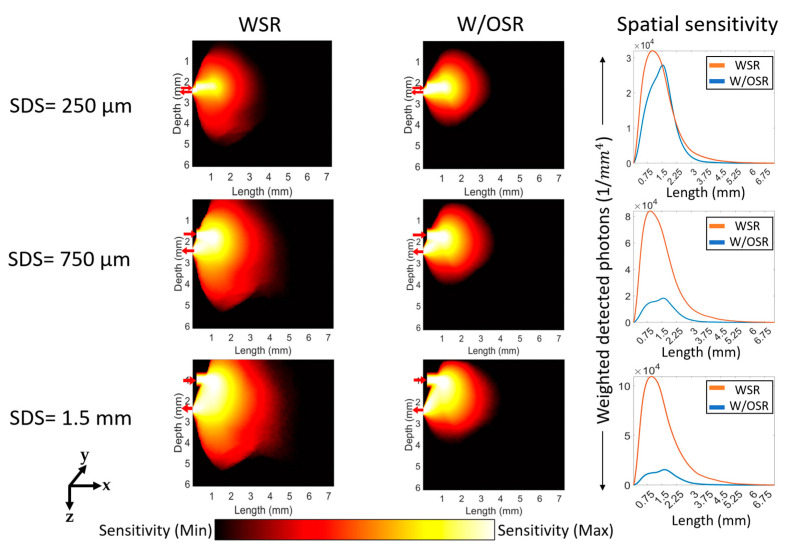
Spatial sensitivity distributions for the reflectance geometry at 633 nm. The labels of ‘SDS = 250 µm’, ‘SDS = 750 µm’, and ‘SDS = 1.5 mm’ refer to the respective three rows and “WSR”, “W/OSR”, and “Spatial sensitivity” refer to the respective three columns. The red arrows in each figure show the respective positions of the optical source and detector, pointing towards and away from the figure. The logscale color bar denotes the sensitivity of absorption within the range of maximum and minimum values.

The Jacobian analysis shown in Figure 7, Figure 8, Figure 9 and Figure 10 was created using perturbation MC with photon numerical replay and Equations (5) and (6) as described in Section 2.3.

Figure 7 illustrates that the number of scattering events that occurred within the tooth volume for 633 nm and 1310 nm wavelengths for the transmittance geometry. The results reveal that the dentin region exhibits the highest number of scattering events, while the pulp region had the lowest number of events for both wavelengths. Additionally, a more detailed view of the distribution of photons scattered along the length for both wavelengths in the transmittance geometry is provided in Figure 7c. As shown in Figure 7, the number of scattering events at 633 nm is consistently higher than that at 1310 nm at all lengths. The maximum scattering events occur at a length of 2.27 mm and 2.22 mm for 633 nm and 1310 nm, respectively. As light penetrated further into the tissue, the number of scattering events decreased.

In Figure 8, the number of scattering events at 633 nm for the reflectance geometry is higher than that at 1310 nm for a short SDS of 0.25 mm. However, as SDS increases, the number of scattering events at 1310 nm is much higher than that at 633 nm (third column in Figure 8). At short SDS, the maximum scattering events occur at a length of 1.68 mm and 1.75 mm (correspond to dentin region) for 633 nm and 1310 nm, respectively. At long SDS of 1.5 mm, the maximum scattering events occur at a length of 1.66 mm and 1.72 mm for 633 nm and 1310 nm, respectively. Beyond a length of 2.25 mm, the number of scattering events at 1310 nm is slightly higher than that at 633 nm. Moreover, as the light goes deeper into the tissue, the number of scattering events decreases.

**Figure 11:**
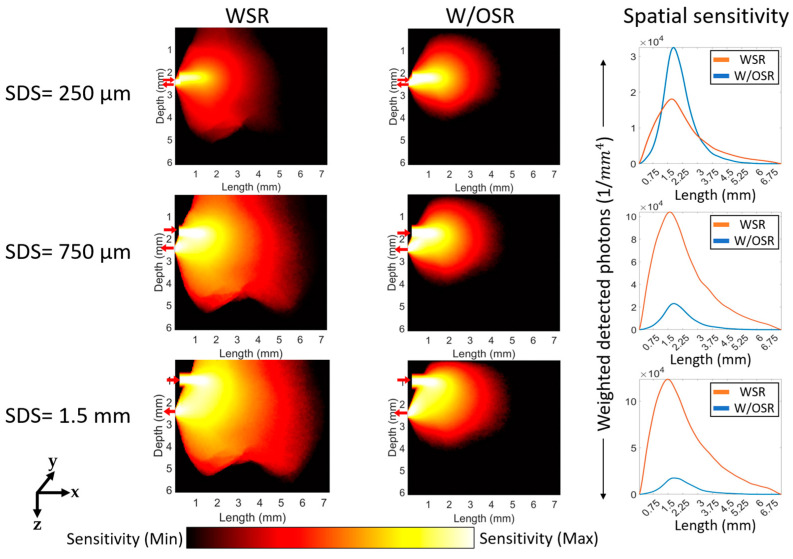
Spatial sensitivity distributions for the reflectance geometry at 1310 nm. The labels of ‘SDS = 250 µm’, ‘SDS = 750 µm’, and ‘SDS = 1.5 mm’ refer to the respective three rows and “WSR”, “W/OSR”, and “Spatial sensitivity” refer to the respective three columns. The red arrows in each figure show the respective positions of the optical source and detector, pointing towards and away from the figure. The logscale color bar shows the sensitivity of absorption within the range of maximum and minimum values.

The sensitivity of the detection system to the changes of localized absorption coefficients was assessed for both transmittance (Figure 9) and reflectance geometries (Figure 10 and Figure 11) under both WSR and W/OSR conditions. The spatial sensitivity was calculated as weighted detected photons [29] from all time gates (1 ns) which reached the detector as described in Section 2.3.

**Figure 12:**
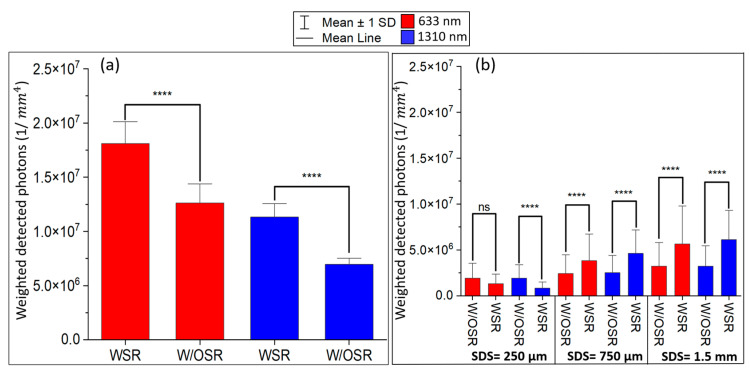
System’s sensitivity to absorption in the transmittance (**a**) and the reflectance geometry (**b**). The sensitivity of the system was defined as the sum of weighted detected photons at each compartment. WSR = with surface reflection, and W/OSR = without surface reflection at boundaries. In the transmittance mode, the detected signal is significantly different between the WSR and W/OSR at two wavelengths. Detected signal is not statistically significant at 250 µm SDS for the reflectance mode at 633 nm (*p*-value = 0.06; Student’s *t* test). The error bars show 1 standard deviation. *p*-value > 0.05 was defined as non-significant (ns) and *p*-value < 0.0001 considered as extremely significant (****).

For the transmittance geometry shown in Figure 9, the system was more sensitive to the changes of localized absorption coefficients at 633 nm than that at 1310 nm. On the other hand, Figure 12a indicates that the spatial sensitivity was significantly higher in WSR compared to W/OSR conditions for 633 nm (1.78 × $10^{7}$ vs. 1.29 × $10^{7}$, *p*-value < 0.0001, Student’s *t* test). Similarly, at 1310 nm, the spatial sensitivity was significantly higher in WSR compared to W/OSR conditions for (1.12 × $10^{7}$ vs. 0.68 × $10^{7}$, *p*-value < 0.0001, Student’s *t* test).

For the reflectance geometry shown in Figure 10 and Figure 11, all SDSs except for 250 µm (third column in Figure 10 and Figure 11) WSR at boundaries resulted in a significantly higher sensitivity compared to W/OSR at boundaries (*p*-value < 0.0001, Student’s *t* test). For both 633 nm and 1310 nm, as SDS increases, the total sensitivity of the system (total detected photons) also increases (Figure 10 and Figure 11).

According to Table 3, the contribution of the pulp region to the total detected signal was greater in the transmittance geometry compared to the reflectance mode at all SDSs, which are 250 µm, 750 µm, and 1.5 mm. Additionally, as the SDSs increases, the contribution of the detected signal from the pulp region also increases, and this increase was higher for WSR than that in W/OSR conditions. In the transmittance mode, the reflection at boundaries resulted in a lower contribution of the pulp region at 1310 nm, whereas at 633 nm, the WSR condition increased the contribution of Pulp by 57% compared to W/OSR (Table 3). 

## 4. Discussion

This article presented a MC model that can aid in evaluating the performance of DOS systems used for dentistry applications operating at 633 nm and 1310 nm wavelengths in both reflectance and transmittance modes. Unlike prior tooth models [11,17], the newly developed model accurately represents the geometry of a second molar tooth and is designed with meshed-based structures for dental tissues such as enamel, dentin, and pulp regions. This precise geometry allows for more reliable and accurate results. The findings showed that to achieve an error rate of less than 1% for detected photons, a minimum of $6\times 10^{8}$ input photons should be simulated. By meeting these requirements, the convergence rate of 0.00004 can be reached, indicating that the developed model is accurate and reliable for photon detection.

In this study, in order to evaluate the device’s performance, the spatial sensitivity of the system was defined via perturbation MC as the integrated weighted detected photons at the detectors [29]. At 633 nm, the system’s sensitivity was significantly higher in the transmittance mode than that in the reflectance mode for both WSR and W/OSR conditions (Figure 12). At 1310 nm, the WSR condition caused the system’s sensitivity to be significantly higher in the transmittance compared with the reflectance (*p*-value < 0.0001; Student’s *t* test). This suggests that the transmittance geometry is generally more sensitive to the detected signal than the reflectance mode, especially with SDS < 1.5 mm. This study has found that the pulp region, which has a blood content of less than 10%, has the least impact on the detected signal compared to other dental compartments at both 633 nm and 1310 nm wavelengths. Surface reflection at boundaries had a positive impact on the system’s sensitivity in both transmittance and reflectance modes. In the transmittance mode optical devices operating at 633 nm and 1310 nm, the contribution of the pulp region was higher than that at 1310 nm when W/OSR conditions were applied to boundaries. On the other hand, the findings indicated that the contribution of the pulp region for the reflectance mode optical devices (750 µm and 1.5 mm SDSs) operating at 1310 nm is significantly higher than that at a 633 nm wavelength for both WSR and W/OSR conditions (*p*-value < 0.01; Fisher’s exact test). Additionally, this research indicated that longer SDSs in the reflectance mode geometry were more likely to detect signals originating from the pulp region than shorter SDSs. This suggests that with surface reflection at boundaries and a 1.5 mm SDS, the contribution of the pulp region at 1310 nm was 4.75 times greater than that at 633 nm. Although a larger SDS can provide higher sensitivity, it may not be practical to have an SDS larger than 1.5 mm in real-world optical imaging systems as it could interact with the gum region or be uncomfortable for patients. In comparing the sensitivity of transmittance and reflectance mode geometries in Figure 12, it was found that the system’s sensitivity for WSR at boundaries at 633 nm in the transmittance mode was significantly higher than the same wavelength in the reflectance mode with the longest SDS (1.5 mm). However, the exact values of the mean optical path length at each wavelength in dental tissue are challenging to calculate and depend on specific imaging conditions in practical applications.

One limitation of the present study is that only two specific wavelengths (633 nm and 1310 nm) were evaluated, and other wavelengths may have different sensitivities and detection capabilities [6,21,31,32]. In future research, it would be expected to expand the current study to include more extensive and diverse samples of teeth to assess the generalizability of the findings to different populations. Finally, the developed geometry and setup parameters (e.g., sensor size and SDSs) are consistent with literature that has used laser Doppler (LD) for dental application [33,34]. This allows for investigating the feasibility of utilizing the presented MC model not only for simulating LD devices but also for optical devices that rely on coherence gating, such as OCT and coherence-gated Doppler (CGD) [35].

## 5. Conclusions

In conclusion, the research findings demonstrated that in the transmittance mode DOS systems, light at 633 nm travels through dental tissues for a longer distance compared to that at 1310 nm. Conversely, in the reflectance mode, the opposite was observed. Additionally, the study revealed that WSR at boundaries can result in higher fluence at 633 nm compared to that at 1310 nm. This study has also shown that dentin is a highly scattering compartment in the tooth structure. Furthermore, DOS systems working in the transmittance mode geometry are more effective at detecting signals from dental tissue compartments than those using the reflectance mode. Moreover, simulation outcomes indicated that in the reflectance geometry, the system is more sensitive to the pulp region at 1310 nm compared to that at a 633 nm wavelength, indicating its potential for diagnosing dental conditions involving the pulp region. Overall, the signal strength of the pulp region was found to be greater in the transmittance mode devices than that in the reflectance mode. These findings are crucial for the improvement and advancement of dental imaging technologies and may lead to more precise and efficient dental diagnosis and treatment.

## Figures and Tables

**Table 1:** The volume fraction used to model the molar tooth.

Tissue Type	Volume Fraction
Enamel	0.44
Dentin	0.52
Pulp	0.04

**Table 2:** Optical properties of the dental tissue compartments used for simulation. It has been assumed that the dental model was surrounded by air.

Tissue Type	$\mu_{a}\;\left({\mathrm{mm}}^{-1}\right)$		$\mu_{s}\;\left({\mathrm{mm}}^{-1}\right)$		g		n
	633 nm	1310 nm	633 nm	1310 nm	633 nm	1310 nm	
Enamel	0.04	0.12	1.5	3	0.7	0.93	1.63 [16]
Dentin	0.30	0.40	26	25	0.93	0.97	1.54 [16]
Pulp	0.035	0.09	10	3.75	0.97	0.85	1.39 [11]

**Table 3:** Contribution of pulp region in the total detected signal. WSR = with surface reflection, and W/OSR = without surface reflection.

Wavelength (nm)	Geometry	SDS (mm)	$\mathrm{Weighted}\;\mathrm{Detected}\;\mathrm{Photons}\;(1/{\mathrm{mm}}^{4})$		Contribution (%)	
			WSR	W/OSR	WSR	W/OSR
633	Reflectance	0.25	$2.67\times 10^{3}$	$1.10\times 10^{3}$	0.196	0.0579
		0.75	$1.09\times 10^{4}$	$2.19\times 10^{3}$	0.273	0.0859
		1.5	$3.07\times 10^{4}$	$6.63\times 10^{3}$	0.515	0.188
	Transmittance		$5.75\times 10^{5}$	$2.55\times 10^{5}$	3.20	2.04
1310	Reflectance	0.25	$8.15\times 10^{3}$	$6.57\times 10^{3}$	0.823	0.329
		0.75	$5.35\times 10^{4}$	$1.34\times 10^{4}$	1.081	0.495
		1.5	$1.46\times 10^{5}$	$5.50\times 10^{4}$	2.241	1.525
	Transmittance		$2.74\times 10^{5}$	$1.37\times 10^{5}$	2.45	2.02

## Data Availability

Not applicable.
